# Statistical Analysis of the Relationship between AETA Electromagnetic Anomalies and Local Earthquakes

**DOI:** 10.3390/e23040411

**Published:** 2021-03-30

**Authors:** Qinmeng Guo, Shanshan Yong, Xin’an Wang

**Affiliations:** 1 The Key Laboratory of Integrated Microsystems, Peking University Shenzhen Graduate School, Shenzhen 518055, China; 1801213282@pku.edu.cn (Q.G.); anxinwang@pku.edu.cn (X.W.); 2 Shenzhen Earthquake Monitoring and Prediction Technology Research Center, Peking University Shenzhen Graduate School, Shenzhen 518055, China; 3 Engineering Department, Shenzhen MSU-BIT University, Shenzhen 518172, China

**Keywords:** statistical analysis, electromagnetic anomalies, earthquakes, superposed epoch analysis

## Abstract

To verify the relationship between AETA (Acoustic and Electromagnetics to Artificial Intelligence (AI)) electromagnetic anomalies and local earthquakes, we have performed statistical studies on the electromagnetic data observed at AETA station. To ensure the accuracy of statistical results, 20 AETA stations with few data missing and abundant local earthquake events were selected as research objects. A modified PCA method was used to obtain the sequence representing the signal anomaly. Statistical results of superposed epoch analysis have indicated that 80% of AETA stations have significant relationship between electromagnetic anomalies and local earthquakes. These anomalies are more likely to appear before the earthquakes rather than after them. Further, we used Molchan’s error diagram to evaluate the electromagnetic signal anomalies at stations with significant relationships. All area skill scores are greater than 0. The above results have indicated that AETA electromagnetic anomalies contain precursory information and have the potential to improve local earthquake forecasting.

## 1. Introduction

Earthquakes are a highly destructive type of natural disaster. They usually occur without warning and do not allow much time for people to react. Earthquakes can destroy a tremendous number of buildings and infrastructures. The number of deaths caused by earthquakes accounts for 54% of the total deaths from various natural disasters worldwide [1]. On average, more than 20 Ms ≥ 7 earthquakes occur each year all over the world. Effective earthquake prediction has an extraordinary significance for reducing casualties and property losses, but it has proven to be a very challenging issue in seismology [2].

A large number of studies have reported various pre-earthquake signals, such as radon anomalies [3,4], atmospheric and ionospheric anomalies [5,6,7], variations of groundwater level [8] and geomagnetic anomalies [9]. There are also many research methods for pre-earthquake signals, such as geophysical methods, large topographic deformation surveys, and geochemical methods. Based on the above research methods and precursor signals, the anomalies of earthquake precursors can be found by comparing the changes of signals before and after the earthquake. Combining the analysis of multiple earthquake cases to find a general rule is an idea to solve the problem of earthquake prediction.

Electromagnetic (EM) phenomena possibly related to earthquakes has also been reported in many articles [10,11,12,13,14,15]. Most studies have focused on the seismic electromagnetic phenomena generated by underground activities before earthquakes or the perturbations in the atmosphere and the ionosphere. Nevertheless, EM precursors are still a highly disputed topic among scientists of different disciplines. Some negative opinions think that any electromagnetic precursor phenomenon is impossible [16]. It is true that the mechanisms for the generation of different EQ (earthquake)-related EM phenomena are not yet fully understood. The complexity of the seismogenic EM systems is obvious, and a great deal of research is necessary before we clearly understand them. The path appears to be long and challenging.

Our team has developed a system that continuously monitors geoacoustic and electromagnetic signals and transmits data to a cloud server in real time via the Internet, and named it AETA (Acoustic and Electromagnetics to AI) [17]. In previous studies, researchers used different methods to detect anomalies in the AETA electromagnetic disturbance data and found pre-seismic anomalies in studies of the 2017 Ms7.0 Jiuzhaigou earthquake [18,19,20]. To further verify the relationship between AETA electromagnetic anomalies and local seismicity, in this manuscript, statistical studies by superposed epoch analysis method were applied to data from 20 AETA stations that have been in operation for more than three years and have abundant earthquake cases in surrounding area.

In Section 2 we introduce the AETA system including the parameters of the electromagnetic sensing probe, and show the electromagnetic data of EMS (Mount Emei) station. In Section 3, we describe in detail the process of superposed epoch analysis, where PCA was used for anomaly detection and the $E_{s}$ parameter was used for selecting earthquakes. The results of statistical analysis are given in Section 4. Then, we discuss the possibility that AETA electromagnetic signal anomalies are related to earthquakes from the physical point of view in Section 5.1, and explain the feasibility of using the AETA electromagnetic signal anomaly in earthquake prediction from the quantitative point of view in Section 5.2. Finally, we summarize the work of this study.

## 2. AETA Electromagnetic Signals

**Figure 1:**
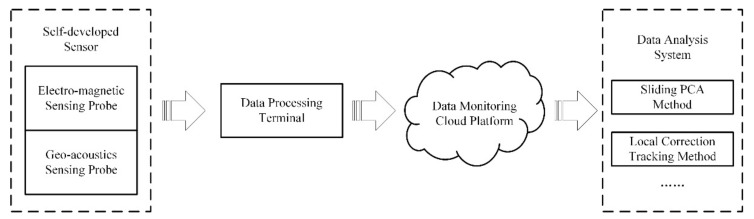
Block diagram of AETA system.

The AETA is composed of a data processing terminal, a geoacoustic sensing probe, an electromagnetic sensing probe (EM probe), a data monitoring cloud platform, and a data analysis system [21]. The probes collect the electromagnetic disturbance and geoacoustic signals, and the data are transmitted to the cloud platform through the network for storage, feature extraction, and anomaly analysis (Figure 1).

The EM probe uses the same principle as the IMSC (search-coil magnetometer instrument) sensor on the French DEMETER satellite and the magnetic sensor on the Chinese electromagnetic satellite [22,23]. Based on Faraday electromagnetic theory, the induced electromotive force was obtained from the magnetic field that changes in the vertical direction, and then, it was processed, including amplification, filtering, and analog-to-digital conversion. The parameters of the AETA EM probe are as follows: 0.1 Hz–10 kHz, 0.1–1000 nT electromagnetic signal, sensitivity of >20 mV/nT@0.1 Hz–10 kHz, 18-bit resolution, and sampling rates of 500 Hz at low frequency and 30 kHz at full frequency [24].

In terms of the equipment reliability, the AETA is a new type of instrument for monitoring earthquake precursors, and it was mass-produced by Zowee Technology Company after it was developed in the laboratory. The AETA was tested for performance, aging, and waterproofing before it left the factory. The reliability and stability of the equipment are guaranteed. Frequency response, sensitivity, noise level, and consistency tests of the EM probe were completed in the magnetic shielding room of the Huailai Remote Sensing Comprehensive Test Station of the Chinese Academy of Sciences. All of the above indicators met the requirement.

**Figure 2:**
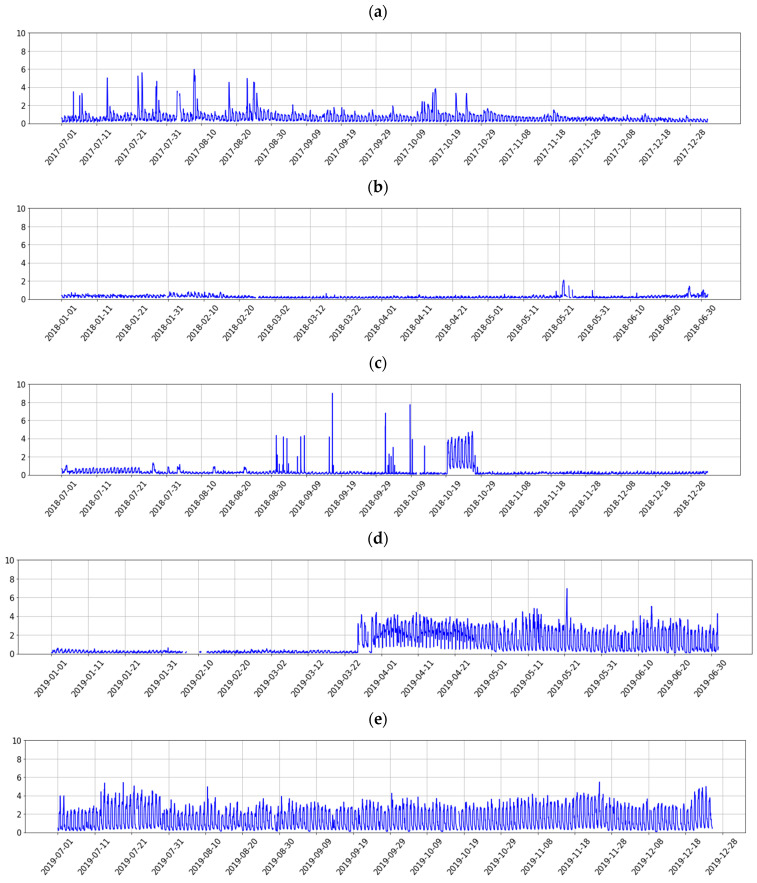

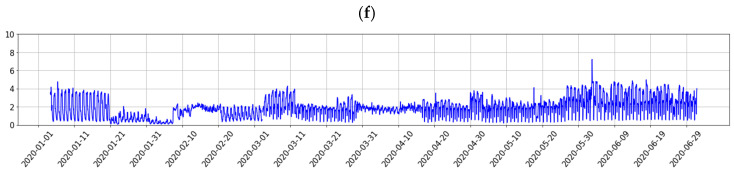
The AETA electromagnetic signals from 1 July 2017 to 1 June 2020 for EMS station; (**a**) from 1 July 2017 to 1 December 2017; (**b**) from 1 January 2018 to 1 June 2018; (**c**) from 1 July 2018 to 1 December 2018; (**d**) from 1 January 2019 to 1 June 2019; (**e**) from 1 July 2019 to 1 December 2019; (**f**) from 1 January 2020 to 1 June 2020.

In this article, we use data from the database, which is obtained by averaging the raw data per minute. The data for EMS station from 1 July 2017 to 1 June 2020 are shown in Figure 2. In each subgraph, the horizontal axis represents the date, the vertical axis represents the signal amplitude in volts (V).

## 3. Superposed Epoch Analysis

Superposed epoch analysis (SEA) reveals the weak but significant signals common to a multitude of events of the same type and averages out signals unrelated to the event and noise. In previous studies, this method has been used to study the relationship between local earthquakes and total electron content anomalies or ultra-low frequency (ULF) geomagnetic anomalies and the relationship between the geomagnetic storms and large-mega earthquakes [25,26,27]. In this study, the SEA method was applied to the statistical study of the seismic electromagnetic phenomena observed by the AETA system. The SEA method involves three steps: anomaly detection, earthquake-related anomalies superposition and random anomalies superposition.

### 3.1. Anomaly Detection

Principal component analysis (PCA) is widely used in data mining and data analysis. PCA can reduce the dimension and extract the interesting features from a data set. PCA was applied to anomaly detection of AETA electromagnetic signals and has achieved good results in the 2017 Jiuzhaigou earthquake case study [28,29]. On this basis, a modified PCA method was proposed.

One data matrix $X=\left(\begin{array}{ccc} x_{1,1} & \cdots & x_{1,n}\\ \vdots & \ddots & \vdots \\ x_{24,1} & \cdots & x_{24,n} \end{array}\right)$ can be formed using *n*-day AETA data, in which a column contains the average of 1 h time intervals in 1 day. We can obtain the covariance matrix *C* of *X*, the eigenvalues of *C* ($\lambda_{1}\geq \lambda_{2}\geq \cdots \geq \lambda_{n}$), and the corresponding eigenvectors stored in a new matrix *E. E* is an orthogonal matrix and satisfies $EE^{T}=I$, where *I* is the unit matrix.

The information for the first *k* principal components can be expressed as

(1) $$\alpha =\frac{\sum_{i=1}^{k}\lambda_{i}}{\sum_{i=1}^{n}\lambda_{i}}\times 100\%,$$

where α is the cumulative contribution rate.

We chose the first *k* eigenvalues with a cumulative contribution rate α of greater than 85% and their eigenvectors $E^{*}=\left(E_{1},E_{2},\cdots ,E_{k}\right)$.

The dimension-reduced matrix *P* is

(2) $$P=XE^{*}$$

The reconstructed matrix $X_{p}$ is

(3) $$X_{p}=PE^{*T}$$

First, the data matrix *X* is constructed by the data of the target date and the data of *n*-1 days before the target date to calculate the background reference value based on a sliding window. The last column $X_{ref}$ of the reconstructed matrix $X_{p}$ is the background value for the target date. In this study, the sliding window length was set to 27 days, which is the solar radiation period in order to reduce the interference from solar activity [30].

To detect the anomaly and reduce the impact of ambient noise, a reasonable tolerance should be scientifically determined. The background value $X_{ref}$ is subtracted from the real value $X_{true}$ and the result is a vector with dimension 24 × 1. The absolute value of each element in this result is sorted in ascending order, and the 23rd value is defined as the tolerance ∆. We obtain the upper and lower bounds as

(4) $$\left\{\begin{array}{c} up=\;X_{ref}+\;∆\;\\ low=\;X_{ref}-∆ \end{array}\right.$$

To eliminate the effect of amplitude differences between different stations, the abnormal value is defined as

(5) $$∆X_{abn}=\left\{\begin{array}{c} \frac{X_{true}-up}{X_{true}},\;X_{true}>up\;\\ \;\;0,\;low<X_{true}<up\;\\ \frac{low-X_{true}}{X_{true}},\;X_{true}<low \end{array}\right.$$

**Figure 3:**
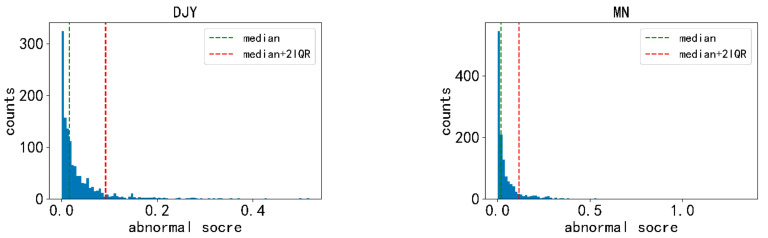
The distribution of the abnormal scores for DJY and MN stations.

Then, we chose the maximum value from the 24 $∆X_{abn}$ per day to indicate the extent of the day’s anomaly, which is defined as the abnormal score. If the abnormal score is directly used for statistical analysis, when the anomalies of an earthquake event are much higher than those of other earthquake events, whether this earthquake event is added to the calculation will have a great impact on the statistical results. Therefore, the abnormal score is transformed to 0 or 1. It is shown in Figure 3 that the distribution of the abnormal scores for DJY and MN stations is skew. The other stations are similar. Generally, the threshold is defined as median + 1.5IQR for the skew distribution. In order to obtain a more robust expression, the threshold is defined as median + 2IQR in this paper. The abnormal score was encoded as 1 if the value exceeded the corresponding median + 2IQR, otherwise it was encoded as 0.

### 3.2. Earthquake-Related Anomalies Superposition

Before introducing the method of earthquake-related anomalies superposition, we first determine the criterion for choosing earthquakes. The magnitude is the reflection of the energy released by an earthquake. The earthquake magnitude difference is twice, the corresponding energy differs by a thousand times. The energy is gradually attenuated from the epicenter. According to this theory, the influence of an earthquake on a station depends on the magnitude and epicentral distance. That is the $E_{s}^{\prime }$ parameter which is expressed in Equation (6). There may be more than one earthquake occurring near a station on the same day. Therefore, the influence of all earthquakes for a station is the sum of each $E_{s}^{\prime }$ and represented in Equation (7) [31].

(6) $$E_{s}{}^{\prime }=\frac{10^{4.8+1.5M}}{r^{2}}$$

(7) $$E_{s}=\sum_{1day}E_{S}{}^{\prime }$$

*M* and *r* are the magnitude of the earthquake and the epicentral distance, respectively.

To make our work consistent with previous studies, earthquake events occurring within 500 km of the station and with an $E_{s}$ index greater than $10^{7}$ were considered [32,33]. According to above two criteria, the list of studied earthquakes was determined. We assumed that there are *N* earthquake events. For each earthquake event, we created an array of encoded abnormal scores for 30 days before and after its occurrence day. Then, we got a two-dimensional array with dimension *N* × 61. Next, we summed this array along the *Y*-axis and got a result with dimension 1 × 61. So far, we have obtained the SEA result in terms of the seismic electromagnetic anomaly.

### 3.3. Random Anomalies Superposition

To evaluate the statistical significance, we randomly picked *N* days within the entire analyzed period of the monitoring station instead of *N* earthquake days. For *N* days, the same procedure as described in Section 3.2 was performed to obtain the expected background values of SEA. To minimize the contingency, we repeated random SEA tests for 30,000 times and calculate the mean (random-mean) and the corresponding standard deviation (σ).

## 4. Results

**Figure 4:**
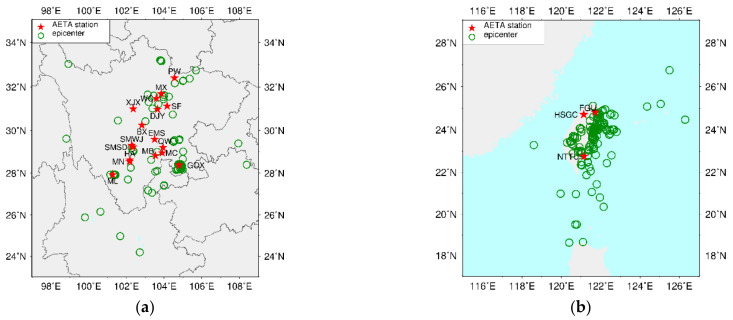
Spatial distributions of major earthquakes with E_s_ > 10^7^ around each selected station: (**a**) Stations in Sichuan; (**b**) Stations in Taiwan. The red star indicates the location of each station and the green circle represents earthquakes.

To perform a statistical study of the AETA electromagnetic anomalies detected by a station, two important things are continuous long-term monitoring data and a certain number of earthquake events. The selected station should be in operation for more than 3 years and the number of earthquake events that satisfied the criterion described in Section 3.2 within the analyzed period should not be less than 20. Therefore, 20 AETA stations were selected as the research objects of this study and their detailed information is given in Table 1. In order to analyze as many earthquake events as possible, the analyzed period for statistical testing at each station is from the month following the station’s installation time to June 2020. The specific analyzed period of each station is shown in the Analyzed period column. The number of studied earthquake events in the analyzed period for each station is given in the EQ num column. The locations of 20 AETA stations and surrounding earthquake events are shown in Figure 4.

**Figure 5:**
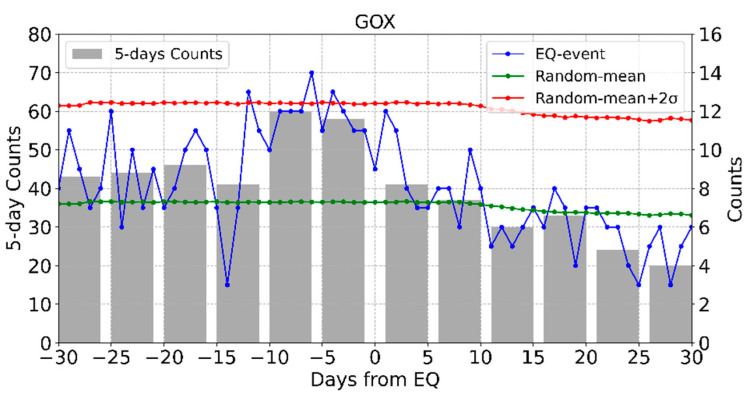
The results of superposed epoch analysis (SEA) for GOX station.

The SEA method was applied to perform statistical studies of the AETA electromagnetic anomalies at 20 selected stations. The accuracy of the statistical results for each station was not affected although the same earthquake may be detected by several stations as shown in Figure 4, because an earthquake is only counted once for each station. Taking GOX station as an example, we have performed statistical analysis on the AETA electromagnetic anomalies observed from February 2017 to June 2020. Figure 5 shows the results for GOX station. Generally, the day of the earthquake is taken as the coordinate 0 point, and the abscissa range is −30 to 30 days. The gray bar shows the counts of anomalies for every 5 days with their values given by the left vertical axis. The blue, green, and red lines show the counts of the AETA electromagnetic anomalies related to earthquake events, random-mean, and random-mean + 2σ, respectively. Their values are given by the right vertical axis. The value of a certain day on the blue line also represents the number of earthquakes that followed the occurrence of an anomaly on that day. Generally, if the earthquake events and the AETA electromagnetic anomalies have no relationship, the counts probably distribute randomly and may not exceed random-mean + 2σ. On the other hand, if the count on a certain day exceeds random-mean + 2σ, the AETA electromagnetic anomalies on this day will have statistical significance. This suggests that the AETA electromagnetic anomalies on this day could be associated with earthquakes. In Figure 5, the result of daily counts 12, 6 and 4 days before the earthquake are significant for GOX station. The value of 5-days counts of 6–10 days before the earthquake is very high. Before an earthquake event, there are clearly higher probabilities of electromagnetic anomalies than after the event. These results are highly suggestive of the relationship between the local earthquake events and the electromagnetic anomalies at GOX station.

**Figure 6:**
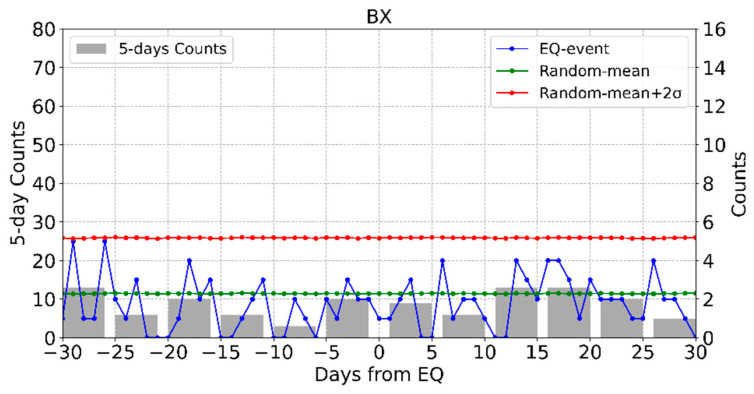
The results of superposed epoch analysis (SEA) for BX station.

The times when the results of daily counts are significantly high at all stations are summarized in Table 2. The results of HSGC, BX, QW and WC stations are a blank. The results of SEA for BX station are shown in Figure 6. The daily counts of BX station were close to but did not exceed random-mean + 2σ in some days. The daily count rate of earthquake-related anomalies is higher than that of random anomalies, but unfortunately it does no show a significant relationship between local earthquakes and the electromagnetic anomalies. Another three stations are similar. The other stations have significant relationship. Meanwhile, the timing at which daily counts are significantly high varies from station to station. 

The generation mechanism of seismic electromagnetic signals is still being explored. The electromagnetic signal may be affected by various environmental factors during its propagation. The phenomenon that some observation stations do not respond strongly to observation signals exists in many earthquake detection systems [34]. It is difficult for stations located in different geographical locations to acquire exactly the same electromagnetic signals. In this study, 80% of selected stations have a highly suggestive of the relationship between local earthquakes and signal anomalies. This shows to a certain extent that electromagnetic signals monitored by AETA stations are useful and contain some information about local earthquakes. Furthermore, the anomalies were more likely to appear before the earthquakes than after them. These results indicate that AETA electromagnetic anomalies have a great potential for serving as a reference for local earthquake prediction.

## 5. Discussion

### 5.1. AETA Electromagnetic Signals

**Figure 7:**
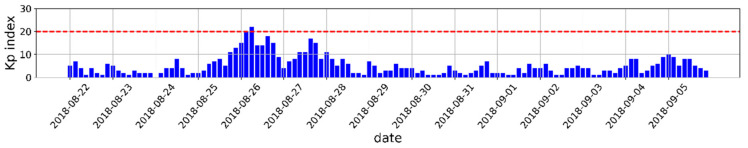
The Kp index before and after geomagnetic storm on 26 August 2018.

**Figure 8:**
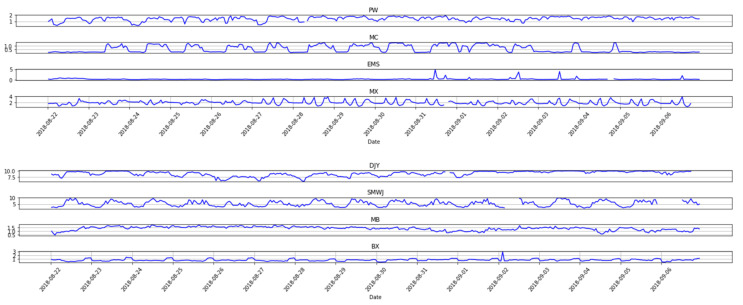
The AETA electromagnetic signals before and after the geomagnetic storm on 26 August 2018 for PW, MC, EMS, MX, DJY, SMWJ, MB, and BX stations.

AETA electromagnetic sensing probe is based on Faraday’s law of electromagnetic induction. Lightning, geomagnetic storms and power frequency signals will inevitably affect the electromagnetic field in the environment. Firstly, the frequency of the AETA electromagnetic signals is less than 200 Hz. However, lightning interference is mainly composed of electromagnetic signals with a frequency of 3 kHz to 30 kHz. Since their frequencies do not overlap, the impact of lightning on the AETA electromagnetic signal is almost negligible. Secondly, geomagnetic storms are a global and violent disturbance phenomenon of the earth’s magnetic field. Figure 7 shows the Kp index before and after the geomagnetic storm on 26 August 2018. The Kp index is used to describe the intensity of geomagnetic disturbances every three hours a day and its value is 0, 0+, 1−, 1, 1+, ..., 9−, 9, a total of 28 levels. In Figure 7, we use 0–27 to indicate the intensity of geomagnetic activity. When the Kp index is higher than 7− (which is equal to 20 in Figure 7), it is considered that a large geomagnetic storm has occurred. Figure 8 shows the electromagnetic data of some stations selected in this paper before and after the same geomagnetic storm. On this day, there was no significant change in the waveform and amplitude of the AETA electromagnetic signal. Finally, there are power frequency signals in AETA electromagnetic signals. Through the analysis of AETA electromagnetic raw data, the power frequency signal exists as a carrier signal. Most of the AETA stations are located near a station deployed by the China Earthquake Administration, and others are located in governments, schools, etc. It can be inferred that the power frequency noise in the environment is relatively weak and stable. Besides, the changes of AETA electromagnetic signals have different forms, such as sudden up and down change, disappearance and appearance of daily periodic signals. Therefore, we infer that the AETA signal change does not reflect the above three interference factors, but other sources, which may be related to potential earthquakes. 

The AETA electromagnetic sensing probe detects changes in the magnetic field by magnetic induction, which is related to the free charges around it. There is no clear conclusion about the generation mechanism of the seismic electromagnetic signal. It is generally believed that seismic electromagnetic signals are related to the rock fracture caused by the accumulation of underground stress. In the process of rock rupture, friction generates heat radiation, piezoelectric effect, or charge separation on the fracture surface generates a strong electric field, which ionizes air molecules in weak areas of the earth’s crust or cracks in active faults. The physical meaning of the AETA electromagnetic sensing probe monitoring data is consistent with the theory of seismic electromagnetics. Therefore, we believe that AETA electromagnetic anomalies have a high possibility to be related to local earthquakes.

### 5.2. Prediction Efficiency Based on AETA Electromagnetic Anomalies

For most of the AETA stations, the electromagnetic anomalies a few days before earthquakes have a clear statistical significance. These anomalies can be used to forecast subsequent earthquakes. If an anomaly occurs, a prediction that an earthquake will happen in the next few days will be made. A modified version of Molchan’s error diagram was used to test the feasibility of simple earthquake forecasting based on AETA electromagnetic anomalies [35]. 

There are two concepts involved in the description of the earthquake prediction: the lead time of the alarms (*D*) and the length of the alarm window (*L*). The lead time is the time interval between the occurrence of the anomaly and the start of the alarm, and the alarm window is the duration of the alarm. Given the above results, the values of *D* and *L* vary from station to station. When the anomaly arises on Day *i*, it is assumed that an earthquake with *E_s_* ≥ $10^{7}$ will occur between Day *i + D* and Day *i + D + L* − 1, and this time period is defined as the alarm days. If an earthquake with *E_s_* ≥ $10^{7}$ occurs during the alarm days, it will be counted as “predicted”, otherwise it will be counted as “missed”. We set *N* equal to the total number of days of the entire analyzed period, *N*_1_ equal to the number of alarm days, *n* equal to the total number of earthquakes, and *n*_1_ equal to the number of predicted earthquakes. The alarm rate can be expressed as $\tau \left(P^{*}\right)=N_{1}/N$. The accuracy rate can be expressed as $v\left(P^{*}\right)=n_{1}/n$. $P^{*}$ is the alarm threshold, which ranges from the minimum to the maximum of the abnormal scores.

**Figure 9:**
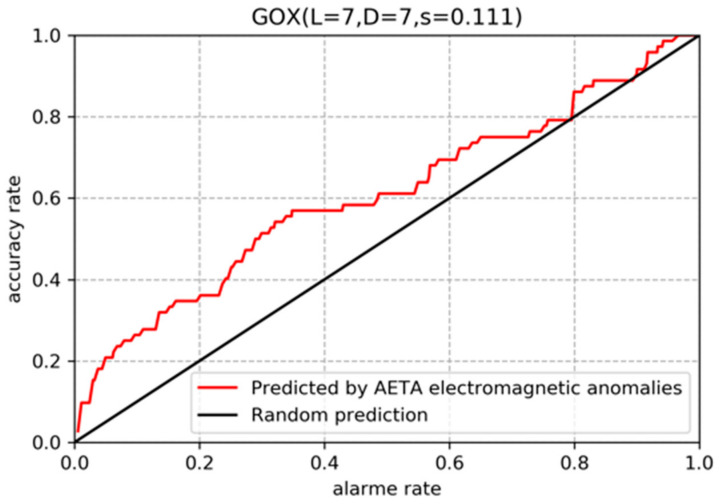
Molchan’s error diagram of prediction at GOX station.

Figure 9 shows Molchan’s error diagram for the GOX station. The diagonal solid black line indicates the prediction by random guess and the solid red line shows the prediction based on the AETA electromagnetic anomalies. The area skill score (s) is defined as the difference between the area under the red solid line and the area under the black solid line. The area skill score of GOX station is 0.111 which indicates that the earthquake predictions based on the AETA electromagnetic anomalies are more effective than random guess. The area skill scores of all 20 stations are summarized in Table 3. The area skill scores of the first 16 stations which have significant relationship are greater than 0. The later 4 stations have no significant relationship and their area skill scores are smaller, some of them are even less than 0. This indicates that the AETA electromagnetic anomalies have the potential to allow effective earthquake prediction especially at stations with significant relationship. Proposing a well-considered and effective earthquake prediction method based on AETA electromagnetic anomalies is well worth exploration in the future.

## 6. Conclusions

In this paper, we investigated the relationship between the AETA electromagnetic signals and local earthquakes. A modified PCA was applied to detect the anomalies and SEA was used for statistical research. The results suggest that the anomalies are more likely to appear before earthquakes at most stations. Moreover, we analyzed the feasibility of earthquake prediction based on AETA electromagnetic anomaly. The results of Molchan’s error diagram showed that AETA electromagnetic anomalies can bring information gain to improve the prediction effectiveness compared to random guess.

There is still a need for long-term exploration because the question of earthquake prediction is complex and difficult. The method of short-term earthquake prediction based on AETA electromagnetic signal anomaly is worth studying in the future.

## Figures and Tables

**Table 1:** Information for 20 selected stations.

No.	Station	Location (E, N)	Installation Time	Analyzed Period	EQ Num
1	DJY	103.65°, 30.98°	27 December 2016	1	29
2	SMWJ	102.28°, 29.31°	19 December 2016	1	26
3	SMSD	102.35°, 29.23°	18 December 2016	1	25
4	MN	102.17°, 27.4°	15 December 2016	1	24
5	FGU	121.72°, 24.82°	13 January 2017	2	98
6	GOX	104.79°, 28.38°	13 January 2017	2	72
7	NTTU	121.15°, 22.75°	13 January 2017	2	101
8	HSGC	121.14°, 24.71°	13 January 2017	2	89
9	MB	103.54°, 28.83°	12 April 2017	5	27
10	ML	101.27°, 27.93°	20 April 2017	5	30
11	HA	102.18°,28.62°	11 May 2017	6	20
12	BX	102.82°, 30.25°	5 June 2017	7	25
13	PW	104.55°, 32.41°	7 June 2017	7	23
14	QW	103.94°, 29.21°	8 June 2017	7	27
15	MX	103.85°, 31.69°	13 June 2017	7	26
16	WC	103.59°, 31.48°	13 June 2017	7	24
17	SF	104.16°, 31.13°	5 June 2017	7	26
18	EMS	103.5°, 29.59°	5 June 2017	7	24
19	MC	103.9°, 28.96°	11 June 2017	7	27
20	XJX	102.36°, 31°	5 June 2017	7	23

Note: In the analyzed period column, 1: 1 January 2017–30 June 2020, 2: 1 February 2017–30 June 2020, 5: 1 May 2017–30 June 2020, 6: 1 June 2017–30 June 2020, 7: 1 July 2017–30 June 2020.

**Table 2:** The results of SEA for selected stations.

No.	Station	Significantly Anomaly Period
1	DJY	13 days before
2	SMWJ	2 days before
3	SMSD	11 days before
4	MN	23 days before, 20 days before, 17 days before, 6 days before, 15 days after, 16 days after, 23 days after
5	FGU	13 days before, 10 days before, 9 days before, 6 days after, 23 days after, 26 days after
6	GOX	12 days before, 6 days before, 4 days before
7	NTTU	0 days before, 7 days after
8	HSGC	-
9	MB	5 days after
10	ML	8 days before
11	HA	14 days before
12	BX	-
13	PW	8 days before, 1 day before, 1 day after, 4 days after, 12 days after
14	QW	-
15	MX	22 days before, 6 days before
16	WC	-
17	SF	4 days after
18	EMS	19 days before, 18 days before, 2 days before, 0 day before
19	MC	5 days before
20	XJX	12–13 days after, 15–21 days after, 23–24 days after

**Table 3:** The area skill score of all 20 AETA stations.

No.	Station	Area Skill Score	No.	Station	Area Skill Score
1	DJY	0.035	11	PW	0.136
2	SMWJ	0.044	12	MX	0.034
3	SMSD	0.114	13	SF	0.156
4	MN	0.162	14	EMS	0.087
5	FGU	0.104	15	MC	0.064
6	GOX	0.111	16	XJX	0.078
7	NTTU	0.074	17	BX	−0.031
8	MB	0.146	18	WC	0.032
9	ML	0.064	19	QW	−0.02
10	HA	0.091	20	HSGC	0.039

## Data Availability

The data presented in this study is available from the authors upon reasonable request.
